# Comparison of the modified Smith-Petersen (S-P) and ilioinguinal (I-I) approaches for periacetabular osteotomy in adult developmental dysplasia of the hip: a retrospective study

**DOI:** 10.1186/s13018-021-02255-2

**Published:** 2021-02-24

**Authors:** Rui Luo, Guomin Li, Bo Li, Ruyin Hu, Yankun Li

**Affiliations:** Department of Orthopaedics, Guizhou Provincial People’s Hospital, No. Zhongshandong Rd, Nanming District, Guiyang, 550002 Guizhou Province China

**Keywords:** Modified Smith-Petersen approach, Ilioinguinal approach, Periacetabular osteotomy, Adult developmental dysplasia of the hip, Retrospective study

## Abstract

**Background:**

Adult developmental dysplasia of the hip is an untreated congenital hip dysplasia that results in adult hip pain. One of the usual and effective methods for the treatment of this condition is periacetabular osteotomy. However, which approach is better between the modified S-P and the I-I approaches is still unclear and controversial.

**Method and materials:**

We retrospectively assessed our experience with the modified S-P and the I-I approaches by inquiring and evaluating intraoperative blood loss, postoperative radiographic material, postoperative function of the hip, and related complications from July 2014 to January 2019.

**Results:**

A total of 61 patients with adult developmental dysplasia of the hip were enrolled, and 33 patients were divided into a modified S-P group and 28 patients were divided into I-I group. The operation time and blood loss of group I-I were higher than that of group modified S-P. Other clinical and radiographic indexes showed no statistical significance between group the modified S-P and I-I groups.

**Conclusion:**

There is no significant difference in the improvement of the function of the hip at the post-operation stage, but group I-I may require more operation time and blood loss at the intra-operation stage.

## Introduction

Developmental dysplasia of the hip (DDH), or congenital hip dislocation (CDH), is a common abnormality in neonates with multiple abnormal morphological features such as the abnormal matching and inclusion between the acetabulum and the femoral head [1, 2]. The etiology of DDH includes inheritance and intrauterine environmental factors, such as family genetic history, female gender, first child, hip delivery, oligohydramnios, torticollis, and lower extremity deformities [3, 4]. Most neonates with DDH can be diagnosed and treated at early age; however, a part of neonates born in the countryside or economically backward areas do not carry out screening of DDH, and many patients with DDH visit orthopedics clinic in young adulthood [5].

Adult developmental dysplasia of the hip is an untreated congenital hip dysplasia that results in adult hip pain and characterized by morphological anomalies of the hip joint that include acetabular dysplasia, decreased acetabular coverage of the femoral head, excessive femoral anteversion, an increased neck-shaft angle, and a shortened femoral neck [6]. The incidence of adult developmental dysplasia of the hip is approximatively 1 to 10%, and the male to female ratio approximates 1 in 6, with 25% of patients in the world having a family history of DDH [7]. In China, the incidence is remarkably lower than other countries, with about a percentage of 0.07 to 1.75%; however, in the northern area, this percentage is higher than the southern part [8].

These morphological abnormalities result in abnormal joint stress that leads to subsequent labral tears and degradation of the articular cartilage, suggesting that the secondary hip osteoarthritis develops at an early age [9]. To correct the deficient and misoriented coverage of the femoral head, and to restore the normal mechanics of the hip, while relieving the pain of hip, one of the usual and effective methods of treatment is the periacetabular osteotomy (PAO), famously known as Bernese PAO, which was developed in 1983 and first described by Ganz et al. in 1988 [10]. Bernese PAO is a polygon-shaped juxta-articular osteotomy, which can obtain large corrections, and in all directions, while protecting the sciatic nerve and permitting mobilization at early post-operation [11]. There are two surgical approaches that can be used in PAO: the I-I and the modified S-P approaches. Usually, most orthopedists prefer to use the modified S-P approach, since it has relatively lower rate of vascular and neural complications when compared to the I-I approach [12–14]. However, the controversy on which approach is better between the modified S-P and the I-I approaches is still unclear and ununified. This retrospective study aims at exploring elaborate this controversy by comparing the modified S-P and the I-I approaches for periacetabular osteotomy in the adult developmental dysplasia of the hip.

## Materials and methods

### Patients

This retrospective study was approved by the ethics committee of Guizhou Provincial People’s Hospital. All periacetabular osteotomies were performed at our institution from July 2014 to January 2019. In this study, we retrospectively assessed our experience with the modified S-P and the I-I approaches by inquiring and evaluating intra-operative blood loss, post-operative radiographic material, post-operative function of the hip, and related complications. The modified S-P and I-I approaches were used in successive time periods with no selection of patients, and all operations were performed by a senior orthopedicians (B. Li). All included patients were regularly followed, and the period of follow-up was no less than 1 year.

### Study design

**Fig. 1:**
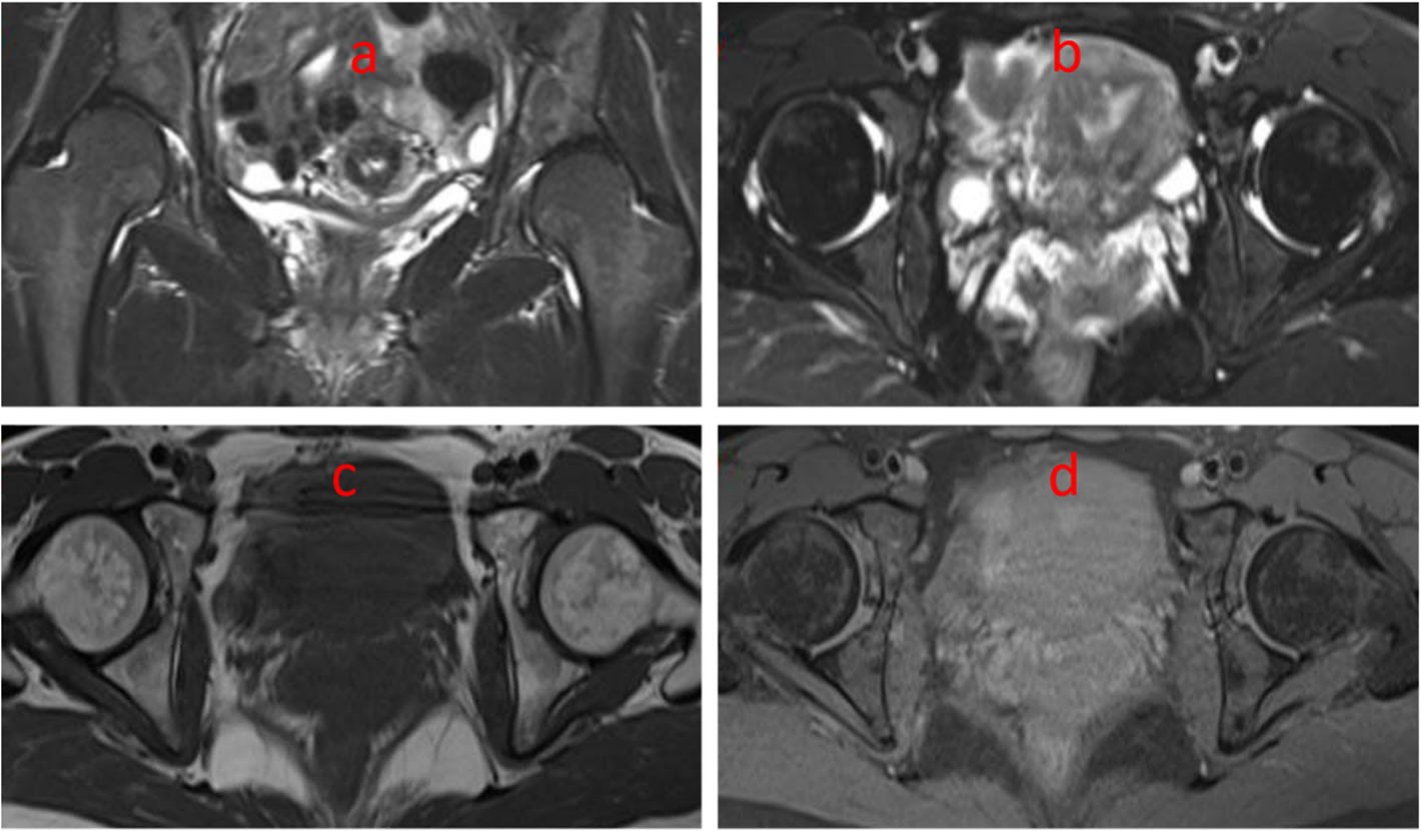
Magnetic resonance images of the hip of a patient with DDH. **a** T2 and coronal plane. **b** T2 and transverse plane. **c**, **d** T1 and transverse plane

The inclusion criteria were as follows: (1) diagnosed adult developmental dysplasia of the hip with painful acetabular dysplasia, (2) age 16–45 years, (3) with closed epiphyseal plates, (4) a center-edge (CE) angle of Wiberg of < 25°, (5) maintained a good range of motion, and (6) Crowel I–III and no or early signs of osteoarthritis (Tönnis grades 0–II) Fig 1. The exclusion criteria were as follows: (1) procedures performed using other approaches, (2) incongruency between the femoral head and the acetabulum, (3) complete dislocation with a secondary acetabulum, and (4) an advanced osteoarthritis (Tönnis grade > III).

### Surgical procedure

**Fig. 2:**
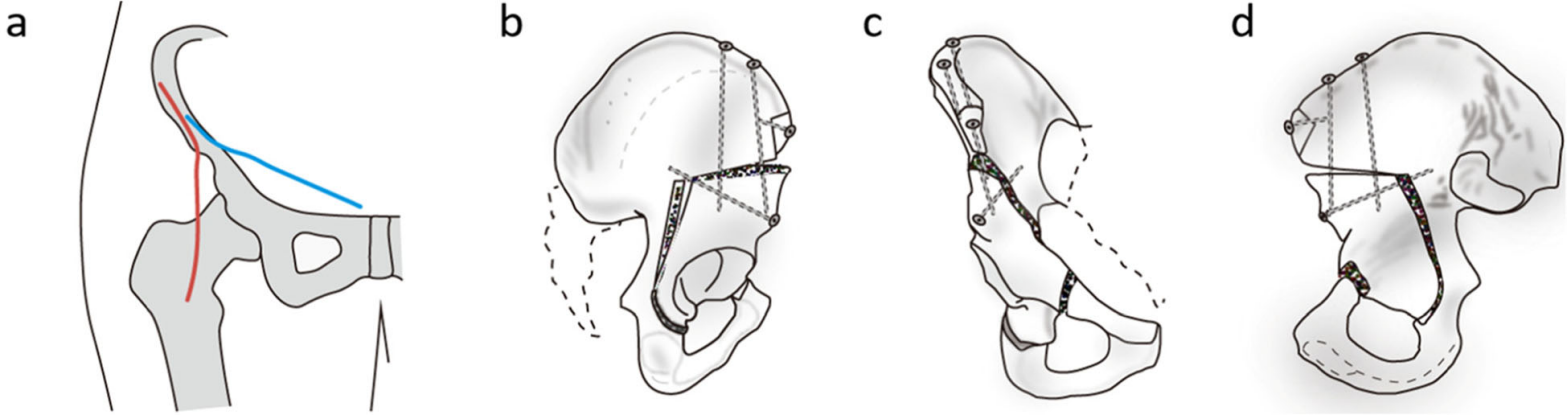
**a** Incisions of the modified Smith-Petersen (red line) and ilioinguinal approach (blue line). **b**–**d** The Bernese periacetabular osteotomy. **b** Four periacetabular osteotomies and a controlled fracture. **c** Fixation of the reoriented fragment. **d** The posterior column of the true pelvis remains intact maintaining stability

The surgery was performed under general anesthesia and the patients were placed into supine position. Intraoperative fluoroscopy was used to control the level of the osteotomies and reorientation of the acetabulum. The surgical incision is shown in the Fig 2a. diagram. The surgical procedure consisted of five osteotomies according to the Bernese periacetabular osteotomy (Fig. 2 b-d).

### Evaluation method

The following parameters were assessed and compared: demographic data, operation time, intraoperative blood loss, complication, clinical data, and radiographic data. Demographic data included age, sex, weight, height, and BMI. Intraoperative blood loss was estimated from the contents of blood in suction bottles and swabs. Operation time was measured from the start of the incision to the closure of the skin. The occurrence of moderate and severe technical, incision, or neurovascular complication was available in the database.

**Fig. 3:**
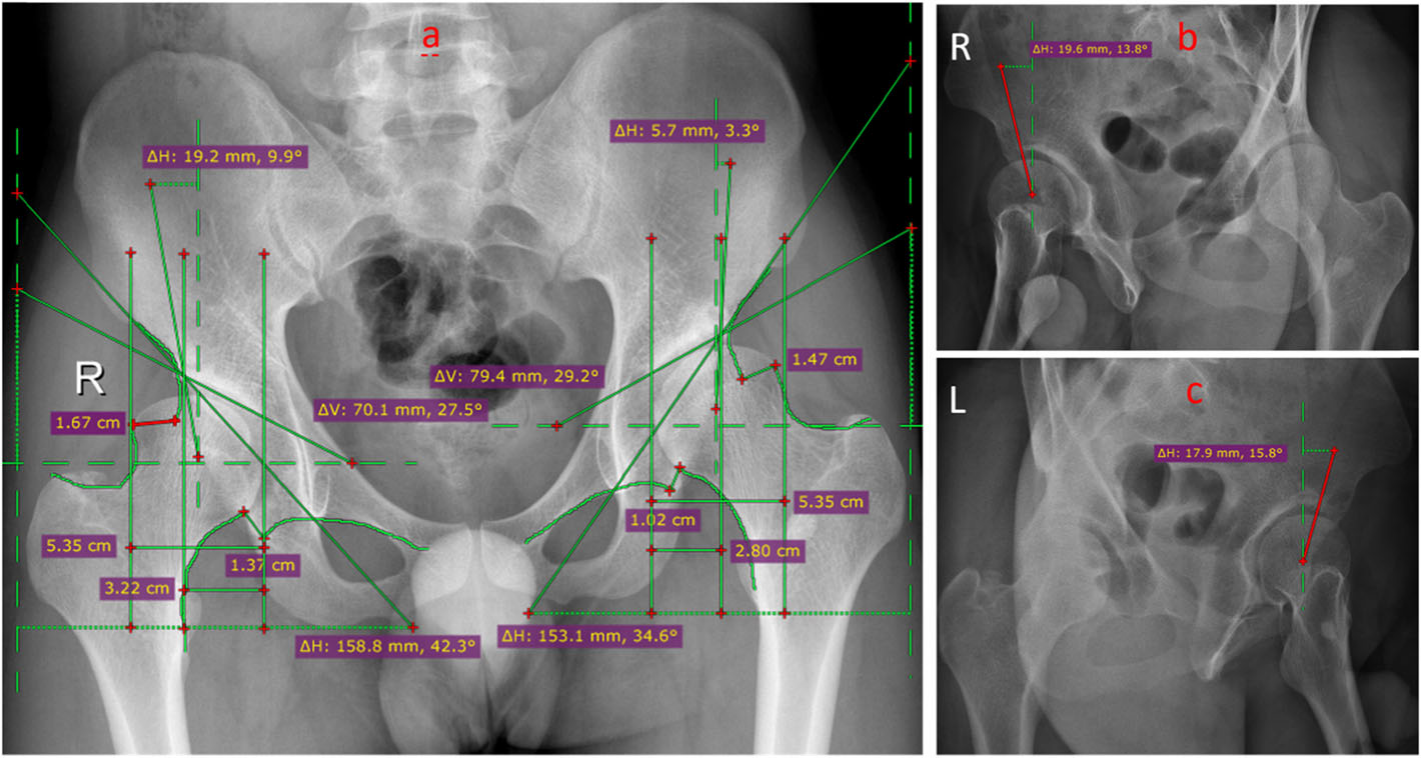
Radiographic images of the hip at pro-operation. **a** Pelvic positive position. **b** Left hip joint 65° oblique piece at pro-operation. **c** Right hip joint 65° oblique piece at pro-operation

**Fig. 4:**
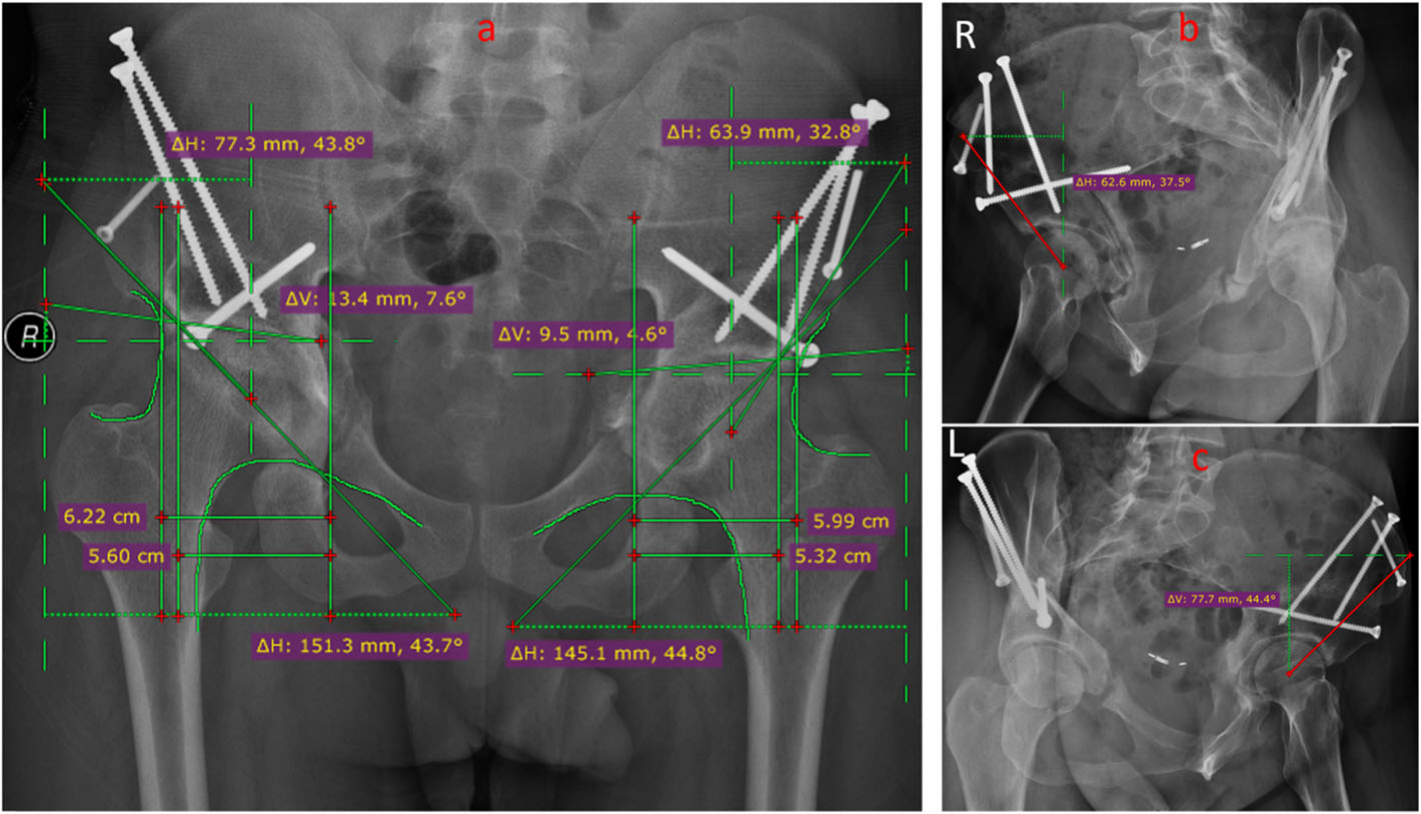
Radiographic images of the hip at post-operation. **a** Pelvic positive position. **b** Left hip joint 65° oblique piece at post-operation. **c** Right hip joint 65° oblique piece at post-operation

The radiographic evaluations were performed with a standard anteroposterior pelvis projection and a false profile view (Fig. 2). The degree of preoperatively hip dysplasia and the reorientation of the achieved acetabulum were assessed by measuring the anterior center-edge angle, the lateral center-edge angle, the total femoral coverage, the sharp angle, the acetabular sign, Shenton’s line intact, Calve’s line intact, and the acetabular index angle (Fig. 3a–c and Fig. 4a–c). The presence and grade of osteoarthrosis was graded according to the criteria of Tönnis (1987). Meanwhile, clinical examinations including the impingement test, the apprehension test, the Trendelenburg sign, and the range of movement (ROM) were used to evaluate the hip function.

### Statistical analyses

All statistical analyses were performed using SPSS, version 24.0, software (IBM Corp, Armonk, NY, USA). All the normally distributed data were presented as means with 95% confidence intervals (CIs), and the non-normally distributed data were presented as median with interquartile range. The independent sample *t* test was used for continuous data in the clinical and radiographic outcomes between the two groups. Paired sample *t* test was used in evaluating changes between pre-operation and post-operation if the variance homogeneity detection is negative, and the Mann-Whitney *U* test was used for continuous data to evaluate pre-operative and post-operative differences in each group or in the same group if the variance homogeneity detection is positive. All binominal data in the two groups were calculated using Fisher’s exact test. A *p* value of less than 0.05 was considered statistically significant [15].

## Results

### Patients

A total of 61 patients with adult developmental dysplasia of the hip were enrolled in the study. The demographic data are shown in Table 1. According to different surgical approaches of PAO, 33 patients were divided into group modified S-P and 28 patients were divided into group I-I. Most patients were female, with the left hip longer than the right hip; however, there were no differences between the two groups in sex, age, side of the hip, weight, height, and BMI. The operation time and blood loss of the group I-I were higher than that of the group modified S-P (operation time: Mann Whitney *U* test, *p* = 0.000; blood loss: 95%CI − 659.47 to − 287.19, *p* = 0.000), and there were two patients who never strain in group modified S-P while there was one patient with incisional infection and the other with a delayed wound healing in group I-I. There were no statistical differences between the two groups (*p* = 0.587).

**Table 1:** Demographic data of the patients

Parameter	Group modified S-P	Group I-I	*p* value
Number of patients (hips)	33 (36)	28 (30)	–
Age at surgery (years)	26.48 ± 8.75	27.14 ± 7.86	0.760
Sex (male to female)	12/21	10/18	0.586
No. of side (right to left)	14/22	12/18	0.563
Weight (kg)	47.79 ± 3.88	47.93 ± 4.09	0.891
Height (cm)	160.03 ± 6.95	160.75 ± 6.94	0.682
BMI (kg/m^2^)	20.41 ± 1.24	20.45 ± 1.20	0.906
Operation time (h)^a^	–	–	0.000
Blood loss (mL)	766.67 ± 368.78	1240 ± 386.50	0.000
Postoperative complication (%)	3 (8.3%)	2 (6.7%)	0.587

The values are given as the mean with the standard deviation in parentheses

^a^Mann Whitney *U* test

### Clinical outcome between group modified S-P and group I-I

The mean function outcomes (poor, fair, medium, good, very good) were similar in the group modified S-P and group I-I at pre-operation and post-operation (pre-operation: *p* = 0.581; post-operation: *p* = 0.992). While comparing pre-operation and post-operation in each group, the function outcomes were remarkably improved (group modified S-P: *p* = 0.000; group I-I: *p* = 0.000). Meanwhile, the mean impingement test, the apprehension test, and the Trendelenburg sign were similar at pre-operation and post-operation. No statistical differences were observed, at pre-operation and post-operation, between the group modified S-P and the group I-I (pre-operation: impingement test-*p* = 0.810, apprehension test-*p* = 0.449, Trendelenburg sign-*p* = 0.737; post-operation: impingement test-*p* = 0.379, apprehension test-*p* = 0.621, Trendelenburg sign-*p* = 0.569), and all pre-operation incidence rates were notably lower than that of pre-operation in both groups (*p* = 0.000).

The ROM of the hip includes flexion, extension, internal rotation, external rotation, abduction, and adduction. There was no evident discrepancy between the group modified S-P and the group I-I at pre-operation and post-operation. Although the degree of extension in the group I-I at pre-operation was bigger than that of the group modified S-P, and the degree of flexion in group modified S-P at post-operation was bigger than that of group I-I, the *p* values were statistically no significant (pre-operation: flexion-*p* = 0.289, extension-*p* = 0.082, internal rotation-*p* = 0.841, external rotation-*p* = 0.250, abduction-*p* = 0.718, adduction-*p* = 0.578; post-operation: flexion-*p* = 0.100, extension-*p* = 0.470, internal rotation-*p* = 0.332, external rotation-*p* = 0.728, abduction-*p* = 0.592, adduction-*p* = 0.428). In the group modified S-P, the ROM of flexion, extension, and adduction had remarkable changes after operation (flexion: *p* = 0.016; extension: *p* = 0.030; adduction: *p* = 0.000), the ROM of internal rotation, external rotation, and abduction were also changed, but the outcomes were not statistically significant (internal rotation: *p* = 0.424; external rotation: *p* = 0.748; abduction: *p* = 0.233). In group I-I, all ROM degrees had changes, but only the degree of flexion, abduction, and adduction was statistically significant (flexion: *p* = 0.002; extension: *p* = 0.076; internal rotation: *p* = 0.092; external rotation: *p* = 0.074; abduction: *p* = 0.027; adduction: *p* = 0.000). All clinical data were shown in Table 2 and Table 3.

**Table 2:** Clinical data for group modified S-P and group I-I

Parameter	Group modified S-P	Group I-I	*p* value
Preoperative			
Function outcome			
Poor	15	10	0.581
Fair	12	15	
Medium	5	2	
Good	3	3	
Very good	1	0	
Impingement test (% of all hips)	18	16	0.810
Apprehension test (% of all hips)	11	12	0.449
Trendelenburg sign (% of all hips)	7	5	0.737
ROM			
Flexion (°)	114 ± 16	110 ± 14	0.289
Extension (°)	4 ± 5	6 ± 4	0.082
Internal rotation (°)	32 ± 21	31 ± 19	0.841
External rotation (°)	36 ± 13	40 ± 15	0.250
Abduction (°)	33 ± 12	32 ± 10	0.718
Adduction (°)	43 ± 14	41 ± 15	0.578
Postoperative			
Function outcome			
Poor	2	1	0.992
Fair	4	3	
Medium	7	6	
Good	13	13	
Very good	10	7	
Impingement test (% of all hips)	3	1	0.379
Apprehension test (% of all hips)	2	2	0.621
Trendelenburg sign (% of all hips)	2	1	0.569
ROM			
Flexion (°)	105 ± 13	100 ± 11	0.100
Extension (°)	7 ± 6	8 ± 5	0.470
Internal rotation (°)	28 ± 19	24 ± 13	0.332
External rotation (°)	35 ± 12	34 ± 11	0.728
Abduction (°)	37 ± 15	39 ± 15	0.592
Adduction (°)	27 ± 11	25 ± 9	0.428

The values are given as the mean with the standard deviation in parentheses

**Table 3:** Clinical data for preoperative and postoperative

Parameter	Preoperative	Postoperative	*p* value
Group modified S-P			
Function outcome			
Poor	15	2	0.000
Fair	12	4	
Medium	5	7	
Good	3	13	
Very good	1	10	
Impingement test (% of all hips)	18	3	0.000
Apprehension test (% of all hips)	11	2	0.000
Trendelenburg sign (% of all hips)	7	2	0.000
ROM			
Flexion (°)	114 ± 16	105 ± 13	0.016
Extension (°)	4 ± 5	7 ± 6	0.030
Internal rotation (°)	32 ± 21	28 ± 19	0.424
External rotation (°)	36 ± 13	35 ± 12	0.748
Abduction (°)	33 ± 12	37 ± 15	0.233
Adduction (°)	43 ± 14	27 ± 11	0.000
Group I-I			
Function outcome			
Poor	10	1	0.000
Fair	15	3	
Medium	2	6	
Good	3	13	
Very good	0	7	
Impingement test (% of all hips)	16	1	0.000
Apprehension test (% of all hips)	12	2	0.000
Trendelenburg sign (% of all hips)	5	1	0.000
ROM			
Flexion (°)	110 ± 14	100 ± 11	0.002
Extension (°)	6 ± 4	8 ± 5	0.076
Internal rotation (°)	31 ± 19	24 ± 13	0.092
External rotation (°)	40 ± 15	34 ± 11	0.074
Abduction (°)	32 ± 10	39 ± 15	0.027
Adduction (°)	41 ± 15	25 ± 9	0.000

The values are given as the mean with the standard deviation in parentheses

### Radiographic differences between the group modified S-P and the group I-I

According to clinical data, all the DDH indexes were similar between the groups in pre-operation and post-operation except a sharp angle in pre-operation (*p* = 0.007). Compared with pre-operation, the total femoral coverage, the anterior center-edge angle, the lateral the center-edge angle, the sharp angle, and the acetabular index were remarkably improved to the normal value of the hip. Meanwhile, Shenton’s line intact and Calve’s line intact were consecutive and smooth lines in post-operation and in all cases. In both groups modified S-P and group I-I, the positive incidence of the crossover sign in post-operation was significantly lower than the one in pre-operation (all of *p* values were 0.000), and there was no significant difference between the groups (pre-operation: *p* = 0.600; post-operation: *p* = 0.463) Tables 4 and 5.

**Table 4:** Radiographic data for group modified S-P and group I-I

Parameter	Group modified S-P	Group I-I	*p* value
Preoperative			
Total femoral coverage (%)	0.60 ± 0.08	0.60 ± 0.09	0.811
Anterior center-edge angle (°)	10.40 ± 3.95	9.79 ± 3.64	0.520
Lateral center-edge angle (°)	11.58 ± 4.66	12.67 ± 4.68	0.346
Sharp angle (°)^a^	–	–	0.007
Acetabular index (°)	28.82 ± 6.89	25.84 ± 7.26	0.092
Crossover sign (% positive)	13(36%)	10(33%)	0.600
Shenton’s line intact (mm)	1.14 ± 0.39	1.08 ± 0.34	0.532
Calve’s line intact (mm)	1.51 ± 0.35	1.46 ± 0.32	0.557
Osteoarthritis score ( Tönnis grade)			
Grade 0	26	21	0.694
Grade 1	7	8	
Grade 2	3	1	
Grade 3	0	0	
Postoperative			
Total femoral coverage (%)	0.89 ± 0.066	0.90 ± 0.056	0.741
Anterior center-edge angle (°)	38.84 ± 3.86	39.57 ± 4.06	0.457
Lateral center-edge angle (°)	35.80 ± 5.57	36.26 ± 5.64	0.741
Sharp angle (°)	40.46 ± 2.40	40.48 ± 2.45	0.979
Acetabular index (°)	7.28 ± 1.33	7.24 ± 1.31	0.887
Crossover sign (% positive)	5(13.9%)	3(10%)	0.463
Shenton’s line intact (mm)	0.00	0.00	–
Calve’s line intact (mm)	0.00	0.00	–

The values are given as the mean with the standard deviation in parentheses

^a^Mann Whitney *U* test

**Table 5:** Radiographic data for preoperative and postoperative

Parameter	Preoperative	Postoperative	*p* value
Group modified S-P			
Total femoral coverage (%)	0.60 ± 0.08	0.90 ± .07	0.000
Anterior center-edge angle (°)	10.40 ± 3.95	38.84 ± 3.86	0.000
Lateral center-edge angle (°)	11.58 ± 4.66	35.80 ± 5.57	0.000
Sharp angle (°)^a^	–	–	0.000
Acetabular index (°)^a^	–	–	0.000
Crossover sign (% positive)	13(36%)	5(13.9%)	0.043
Shenton’s line intact (mm)^a^	–	–	0.000
Calve’s line intact (mm)^a^	–	–	0.000
Group I-I			
Total femoral coverage (%)	0.60 ± 0.95	0.89 ± 0.56	0.000
Anterior center-edge angle (°)	9.79 ± 3.64	39.57 ± 4.06	0.000
Lateral center-edge angle (°)	12.67 ± 4.68	36.26 ± 5.64	0.000
Sharp angle (°)^a^	–	–	0.000
Acetabular index (°)^a^	–	–	0.000
Crossover sign (% positive)	10(33%)	3(10%)	0.057
Shenton’s line intact (mm)^a^	–	–	0.000
Calve’s line intact (mm)^a^	–	–	0.000

The values are given as the mean with the standard deviation in parentheses

^a^Mann Whitney *U* test

.

## Discussion

Periacetabular osteotomy is an effective way to rebuild the acetabular structure, relief the pain after movement, and improve the movement function of the hip. In general, patients whose age ranges from 10 years to 50 years that showed a reduced pain and normal movement function of the hip, a healed acetabular callus line, a good relationship between the head and the acetabular, and a none or mild osteoarthritis are proper indication for PAO. On the contrary, if a patient was much younger or older, or with poor movement function of hip, or poor relationship between the head and the acetabular, or serious osteoarthritis, the outcome of PAO is poor, and therefore, its application is contraindicated [16]. Recently, the ilioinguinal, the Smith-Petersen, and the minimally invasive transsartorial approaches were usually used to perform the periacetabular osteotomy surgery that is a new and relatively safe approach, with minimally invasive transsartorial that can reduce surgical trauma, blood loss, and the duration of surgery. Although more orthopedists consider the minimally invasive transsartorial approach safer than other approaches, it is difficult to perform PAO surgery since the incision is relatively smaller. However, there are some studies that compared the outcome between the ilioinguinal, the Smith-Petersen, and the minimally invasive transsartorial approaches [17–20]. The modified Smith-Petersen approach can achieve the better view and access for optimal manipulation of the acetabular fragment, expose the anterior joint capsule, and facilitate the opening of the joint and exposure of the labrum to treat labral damage, intraarticular cartilage disease, and anterior intracapsular femoral neck deformity. However, the modified Smith-Petersen approach has a high incidence of lateral femoral cutaneous nerve dysesthesias. The ilioinguinal approach is performed on all osteotomies and under direct vision to obtain the best of the deep internal pelvic visibility and the remaining abductor muscles. However, the ilioinguinal approach has a higher risk of serious vascular damage and does not allow an exploration of the hip joint [21–23].

In adult developmental dysplasia of the hip, the abnormal femoral head and acetabular structure and the hip range-of-motion containing flexion, extension, external rotation, internal rotation, abduction, and adduction are inconsistent in normal people. For example, the degree of extension and internal rotation is larger than the normal value, even it is hard to correct to normal degree after rotational acetabular osteotomy (RAO) or periacetabular osteotomy (PAO) surgery [24, 25]. In our study, the hip range-of-motion is a huge discrepancy in healthy people, as the degree of abduction motion is smaller and the degree of adduction motion is obviously larger than in healthy people.

In patients with DDH, the acetabular dysplasia and the total femoral coverage are lower, with the femoral head leading to a shift toward and above the acetabulum leading to luxation and hip instability. It is difficult to determine the center of the hip since the abnormal structure of the hip may cause a little deviation when we measure the angle on an image map especially with the lateral center-edge and the anterior center-edge angles [26]. Meanwhile, most patients with DDH have bilateral leg length discrepancy that leads to pelvic tilt, which affects the limitless of the technology of taking image maps, causes interference, and influences the measuring of angles and indexes of the hip, finally leading to the deviation of results analysis by drawing inaccurate conclusions [27]. In the post-PAO surgery image map, the anatomical sign of acetabular is not obvious or difficult to determine. Radiographic data, in both groups modified S-P and group I-I, may result into errors that influence the final results of the analysis. In our study, we invited three researchers to analyze the radiographic data, and in case of inconsistent results, we discussed the results until drawing same conclusions, or if the disagreement persisted, we sought advice from senior doctors (Tables 4 and 5).

Compared to total hip arthroplasty, periacetabular osteotomy surgery has a higher incidence of complications. As in the front and rear of the acetabular are respectively found the femoral nerve and the sciatic never, PAO surgery is more inclined to injure the nerve and blood vessels that surround the acetabular causing abnormal motion and feeling. The pelvis has a rich blood supply, and PAO has a large damage range that can cause interoperation blood loss in most patients, which would require blood transfusion to restore blood volume. At the same time, acetabular coverage may be incomplete or excessive leading to unsatisfactory function recovery if there is no sufficient preparation during pre-operation [28–30]. In our study, as the incision was long and deep using the ilioinguinal approach, the patient easily acquires an incisional infection, while the surrounding nerve may be pulled by the modified Smith-Petersen approach due to the relatively small incision.

Although periacetabular osteotomy was used to cure adult developmental dysplasia of the hip for many years, the best approach to use among the ilioinguinal, the Smith-Petersen, and the minimally invasive transsartorial approaches is unclear. In our study, we draw a conclusion that there is no significant difference in the improvement of the function of hip at post-operation, but group I-I may take more operation time and more loss of blood in intra-operation. However, there are still several limits. Firstly, this paper is a retrospective study, with many factors that may interfere with the results of the analysis; secondly, the number of patients is smaller, and therefore, there is a need to increase the sample size, and finally, our study is a single-center study, and we need several hospitals to join the study in order to draw a more accurate conclusion in the future.

## Conclusion

In conclusion, the periacetabular osteotomy is an effective way to correct adult developmental dysplasia of the hip, whether using the modified Smith-Petersen or the ilioinguinal approaches. There is no significant difference in the improvement of the function of the hip at post-operation, but group I-I may take more operation time and loss of blood in intra-operation.

## Data Availability

The data and materials contributing to this article may be made available upon request by sending an e-mail to the first author.

## Abbreviations

S-P: Smith-Petersen
I-I: Ilioinguinal
DDH: Developmental dysplasia of the hip
CDH: Congenital hip dislocation
PAO: The periacetabular osteotomy
CE angle: Center-edge angle
ROM: The range of movement
CIs: Confidence intervals
RAO: Rotational acetabular osteotomy
